# Vitamin d and leishmaniasis: Neither seasonal nor risk factor in canine host but potential adjuvant treatment through *cbd103* expression

**DOI:** 10.1371/journal.pntd.0009681

**Published:** 2021-08-16

**Authors:** Clara Martori, Rita Velez, Montserrat Gállego, Ignacio Mesa, Rui Ferreira, Jordi Alberola, Alhelí Rodríguez-Cortés

**Affiliations:** 1 Departament de Farmacologia, Terapèutica i, Toxicologia, Facultat de Veterinaria, Universitat Autònoma de Barcelona, Bellaterra, Spain; 2 Secció de Parasitología, Departament de Biologia, Sanitat i Mediambient, Facultat de Farmàcia i Ciències de l’Alimentació, Universitat de Barcelona, Barcelona, Spain; 3 Instituto de Salud Global de Barcelona (ISGlobal), Barcelona, Spain; 4 Animal Blood Bank, Barcelona, Spain; KU Leuven, BELGIUM

## Abstract

Vitamin D (VitD) deficiency has been shown to be a risk factor for a plethora of disorders. We have shown that dogs with clinical leishmaniasis presented lower VitD serum levels than non-infected dogs, and even lower than those with asymptomatic infection. However, if VitD deficiency is a risk factor to develop clinical leishmaniasis remains to be answered. It is also unknown if VitD participates in *Leishmania* control. First, we retrospectively analysed VitD concentration in serum samples from 36 healthy dogs collected in different periods of the year concluding that there isn’t a seasonal variation of this vitamin in dogs. We also included 9 dogs with clinical leishmaniasis and 10 non-infected healthy dogs, in which we measured VitD levels at the beginning of the study, when all dogs were negative for serology and qPCR, and 1 year later. Whereas non-infected dogs showed no change in VitD levels along the study, those developing clinical leishmaniasis showed a significant VitD reduction at the end of the study (35%). When we compared VitD concentration between the two groups at the beginning of the study, no differences were detected (43.6 (38–59) ng/mL, *P* = 0.962). Furthermore, an *in vitro* model using a canine macrophage cell line proved that adding active VitD leads to a significant reduction in *L*. *infantum* load (31.4%). Analyzing expression of genes related to VitD pathway on primary canine monocytes, we showed that *CBD103* expression was significantly enhanced after 1,25(OH)_2_D addition. Our results show that VitD concentration is neither seasonal nor a risk factor for developing canine leishmaniasis, but it diminishes with the onset of clinical disease suggesting a role in parasitic control. Our *in vitro* results corroborate this hypothesis and point out that VitD regulates infection through *CBD103* expression. These results open the possibility for studies testing VitD as an adjuvant in leishmaniasis therapy.

## Author summary

Vitamin D (VitD), the precursor of the powerful steroid hormone calcitriol, has been widely known to regulate the calcium and phosphate homeostasis. Several studies have shown that VitD plays also an important role on innate immunity. The mechanisms by which VitD modulates the immune function have been studied within different contexts involving multiple pathogens, but not *Leishmania* sp. It is known that VitD strengthens the innate immune system by inducing the expression of anti-microbial peptides in *Mycobacterium tuberculosis-*infected human macrophages. Antimicrobial peptides act on the bacterial wall, increasing the formation of reactive oxygen species, modulating cytokine expression, and inducing autophagy. Immune system plays a key role in leishmaniasis disease control, thus VitD could have a relevant contribution in leishmaniasis. In a previous study, we shown that clinical leishmaniasis is associated with VitD deficiency. This research aims to determine whether vitamin D is seasonal and a risk factor for developing canine leishmaniasis and, also, to study the possible VitD anti-parasitic effect and the expression of genes related to VitD pathway. The results show that VitD in canine leishmaniasis is neither seasonal nor a risk factor for developing clinical disease. However, its role in reducing parasite load suggests that VitD could be an adjuvant in leishmaniasis therapy.

## Introduction

Leishmaniases are a group of neglected vector-borne diseases caused by obligate intracellular protozoan parasites of the genus *Leishmania* (Trypanosomatida: Trypanosomatidae). The disease is considered endemic in tropical and subtropical regions of the Palearctic and Neotropic ecozones, and in ecoregions around the Mediterranean Basin. Human visceral leishmaniasis (VL) can be fatal if left untreated, resulting in 26 000–65 000 deaths per year (World Health Organization, 2019). Canids are the main reservoir and hosts of *L*. *infantum*, the causative agent of zoonotic VL in the Mediterranean Basin [1]. Only in western Mediterranean countries, there is an estimate of 2.5 million infected dogs [2]. Most *Leishmania*-infected humans and dogs show an asymptomatic infection, with only 5–20% of the cases developing the patent disease over a variable period of time [3,4]. The mechanisms that regulate the final outcome of the infection remain unknown, although it appears that asymptomatic infections are associated with a strong specific cell-mediated immunity and a Th1-proinflamatory immune response [5]. Some studies show that Th17 cells act synergistically with the Th1 population to control *L*. *infantum* growth by modulating some key regulatory cytokines [6]. However, exacerbated Th1 and Th17 may be responsible for excessive inflammation and pathology [7,8]. Susceptible individuals develop progressive disease with increasing parasite burden, concomitant to high antibody levels and a progressive Th2-deactivating immune response in the presence of a strong inflammatory reaction [9,10]. Innate immune system plays an important role by promoting the appropriate adaptive cellular immune response against the *Leishmania* infection [11]. Macrophages are central players in innate immune response and the main host cell for *Leishmania* spp.

Several studies have shown the important role that vitamin D (VitD) plays on innate immunity [12–14]. In humans, active VitD can be obtained from a small number of foods, from dietary supplements or photochemically through conversion of provitamin D by UVB rays exposure [15], resulting in a poorer VitD status in non-summer seasons [16]. Previous studies suggest that there would be no seasonal variations in vitamin D levels in dogs due to inadequate cutaneous synthesis [17], but this should be confirmed. The effects of VitD are mediated by its binding to the vitamin D receptor (VDR) which is expressed in several antigen-presenting cells and acts as a transcription factor specifically modulating the expression of many genes [12,18]. In human macrophages, toll-like receptors (TLR) activation by *Mycobacterium tuberculosis* antigens increases the expression of *VDR* and 25-hydroxyvitamin D 1α-hydroxylase (*CYP27B1*) genes, resulting in an over-expression of target genes, including antimicrobial peptides (APM) such as cathelicidins and β-defensins genes [19]. These peptides carry out several anti-microbial functions by increasing the formation of reactive oxygen species, modulating cytokine expression and inducing autophagy [20,21]. In the last decade, other transcriptional regulators that participate in the secondary gene regulatory response to VitD have been also described [22]. Also, it is well established that VitD plays a role modulating adaptive immune response against infection by reducing the production of pro-inflammatory Th1 and Th17 derived cytokines [23,24], IgG secretion and maintaining B-cell homeostasis [25].

Some observational, preclinical, and clinical studies have shown that low levels of VitD increase the risk of developing multiple diseases in humans (cancer, diabetes, autoimmune diseases and infectious diseases such as toxoplasmosis, AIDS, influenza or malaria) [26–32]. However, few studies have investigated VitD and leishmaniasis, with contradictory results depending on the animal model and *Leishmania* species [33–36]. We previously showed, for the first time, that dogs with clinical leishmaniasis and also those with asymptomatic infection presented lower VitD serum concentration than non-infected dogs. Vitamin D concentration were strongly negatively correlated with clinical severity, parasite load, and anti-*Leishmania* IgG levels. However, no association was found between VitD and T-cell response. Therefore, we reported that progression of clinical CanL was strongly associated with VitD deficiency in dogs [37].

The aims of the current study were i) to evaluate if VitD concentration in dogs shows a seasonal variation as it is the case for humans; ii) to investigate whether low VitD concentration is a risk factor for developing canine leishmaniasis (CanL); and iii) to determine if VitD has an anti-parasitic effect in *Leishmania*-infected macrophages and analyze RNA expression of components of VitD pathway.

## Methods

### Ethics statement

The research protocol was submitted to the Ethics Committee on Animal Experimentation (CEEA) of Universitat de Barcelona which, in compliance with national (Royal Decree 1201/2005) and European Union regulations (European Directive 86/609/CE) for projects using animals for research purpose, considered that an ethical approval was not required for this study. The project was also submitted to and approved by ISGlobal Internal Scientific Committee (ISC). All dog owners were informed about the research protocol and signed an informed consent allowing for sample and data collection.

### Dog population included in seasonality study

In order to know if there is a seasonal variation in vitamin D levels in dogs, we included serum samples from thirty-six dogs from different breeds and ages living in Spain, which remained clinically healthy and *Leishmania*-free thorough one year (2016–2017). Serum samples from these dogs obtained in three timing points (February 2016, May/June 2016 and January 2017) were analysed. All dogs were fed commercial dry diet during the entire studied period. No changes in diet were made between timing points.

### Dog population included in longitudinal study

To study the relationship between VitD levels and evolution of *L*. *infantum* infection we included retrospective serum samples from 19 dogs belonging to a longitudinal study of CanL living in a highly endemic area (Spain). Nine of these dogs became *Leishmania* infected, while 10 remained healthy and *Leishmania*-free along the study. This studied population included 60% males and 40% females from different breeds, and ages ranging from 1 to 11 years (4 ± 3.3 years). Samples at Spring times T1 and T2 were analysed.

### Clinicopathological characterization

Clinical signs, clinical chemistry, and haematological values were scored using a clinicopathological score (CPS) as previously described [38]. Briefly, clinical signs compatible with leishmaniasis such as cutaneous lesions, ocular signs, and epistaxis were scored 0–3. Both clinical chemistry and haematological results scored 1 point for each abnormal value. These scores were added to obtain an overall clinicopathological score for each dog.

### Samples

Serum was obtained after centrifugation of peripheral blood at 1600 *g* for 10 min and stored at -20°C until serology and biochemical analyses were performed. Popliteal lymph node samples collected by fine needle aspiration in sterile 0.9% NaCl solution were frozen at -20°C until DNA extraction. Peripheral Blood Mononuclear Cells (PBMCs) were isolated from blood in heparin tubes and preserved in liquid nitrogen until processing.

### Crude total *L*. *infantum* antigen (CTLA)-based ELISA

B cell function was analysed through anti-*Leishmania* antibody concentration using an ELISA technique, as previously described with minor modifications [39]. Briefly, microtiter plates were coated with 2 μg of CTLA per well and sequentially incubated with sera and protein A conjugated to horseradish peroxidase (Pierce). Working dilutions were 1/400 and 1/10 000 for sera and protein A-HRP, respectively. Absorbance values were read at 450 nm in an automatic microELISA reader (Spark 10M, Tecan). Results were expressed in ELISA units (EU), referred to a known positive serum used as a calibrator and arbitrarily set to 1 EU. Cut-off value (mean + 3 SD) for 76 dogs from a non-endemic area was 0.200 OD.

### Real-Time PCR amplification of *Leishmania* DNA

Parasite load was determined by qPCR in lymph nodes. DNA was extracted using the High Pure PCR Template Preparation Kit (Roche). *L*. *infantum* DNA was specifically detected and quantified with a TaqMan qPCR Assay (Applied Biosystems) following a previously reported protocol [40] with some modifications. The qPCR assay was designed to target conserved DNA regions of the kinetoplast from *L*. *infantum* genome. Primer sequences were LEISH-1 5′-AAC TTT TCT GGT CCT CCG GGT AG-3′, LEISH-2 5′-ACC CCC AGT TTC CCG CC-3′, and the TaqMan-MGB probe FAM-5′-AAA AAT GGG TGC AGA AAT-3′- MGB. The thermal cycling profile was 50°C for 2 min, 95°C for 10 min, 40 cycles at 95°C for 15 s, and 60°C for 1 min. Analyses were performed in a StepOnePlus Real Time PCR System device (Applied Biosystems). Each sample plus a negative control was analysed in triplicate. Parasite quantification was performed by comparison with a standard curve generated with *L*. *infantum* DNA extracted from 1 × 10^7^ parasites by using serial dilutions from 10^3^ to 10^−3^ parasites. This technique was sensitive enough to detect 0.001 parasites per reaction with a dynamic range of 10^7^. The median slope of three different standard curves was −3.44, and the qPCR efficiency was 98%. Quantification was linear between 10^3^ and 10^−2^ parasites per reaction tube (correlation = 0.99).

### Determination of Vitamin D levels in serum samples

The prehormone 25-hydroxyvitamin D (25(OH)D) is the most reliable estimate of overall VitD status because this is a stable circulating metabolite of VitD, and its concentration is nearly 1000-fold higher than the biologically active form 1,25-dihydroxyvitamin D (1,25(OH)_2_D). Thus, 25(OH)D levels were assessed in serum samples using a competitive direct enzyme-linked immuno-sorbent assay (IDS 25-Hydroxy Vitamin D Direct EIA kit, Immunodiagnostic Systems Ltd.) according to the manufacturer’s instructions and employing an automatic micro-ELISA reader (Spark 10M, Tecan). The concentration of 25(OH)D in each sample was calculated using a four-parameter logistic curve fit (Prism 5, GraphPad Software), and results were expressed in ng/mL units.

### *In vitro* evaluation of Vitamin D effect on *L*. *infantum* parasite killing

*L*. *infantum* promastigotes of the MCAN/ES/92/BCN-83/MON-1 strain were cultured at 26°C in R15 medium [RPMI 1640 medium (Gibco) supplemented with 15% heat-inactivated fetal bovine serum (FBS) (Gibco), 2% HEPES 1M (Gibco) and 1% 10000 U/mL penicillin with 10 000 μg/mL streptomycin (Gibco)]. Weekly passages were performed. Metacyclic promastigotes for *in vitro* infections were obtained from a 6-days-old stationary culture. DH82 dog macrophages kindly provided by Dr. Javier Moreno (Instituto de Salud Carlos III, Spain). Cells were cultured in R-10 media [RPMI 1640 medium (Gibco) supplemented with 10% FBS (Gibco) and 1% Penicillin/Streptomycin (Gibco)] and kept in a humid atmosphere at 37°C and in 5% CO_2_. The day of the experiment, cells were cultured in a 24-well plate (250 000 cells per well) and left to adhere for 2h. Different concentrations of 1,25-dihydroxyvitamin D (CAS N 250-963-8, Sigma-Aldrich) were added to DH82 cells in triplicate (0.01 μM, 0.1 μM and 1 μM) and plates were incubated for 24 h. After 24 hours, pre-treated DH82 cells were infected with metacyclic promastigotes at a parasite:cell ratio of 5:1, incubated for 24 h and then, washed with 1× PBS to discard non-internalized promastigotes. Cells were treated with trypsin-EDTA 0.05% (Gibco) and plated on microscopic slides by cytocentrifugation (Thermo Scientific Shandon Cytospin 4). Preparations were fixed with methanol and stained with Giemsa 10%. The number of infected macrophages and of intracellular parasites were recorded by direct microscopic count of 200 cells per sample. Values of infected macrophages and parasite burden were expressed as absolute number per 100 macrophages.

### Monocytes isolation

Monocytes were isolated from 2 groups of dogs: a) Nine dogs from the field study, 4 *Leishmania*-infected dogs and 5 non-infected dogs at T2; b) Six healthy dogs whose buffy coats were obtained from the animal blood bank of Spain (Banco de Sangre Animal SL). Buffy coats were obtained by centrifugation (Megafuge 40R, Thermo Scientific) of 450 ml whole blood bags at slow speeds (4000 rpm, 17 min) at 22° C, after which the buffy coat was transferred into an attached satellite bag. First, PBMCs were isolated using Ficoll density gradient method. Samples were diluted (1:3) with 1x PBS, gently layered over 15 mL of Ficoll Paque Plus solution (GE Healthcare) and centrifuged at 400 g for 30 min. The buffy coat cells collected at the interface were washed with 1× PBS and treated with 4 mL of Ammonium-Chloride-Potassium Lysing Buffer (150 mM ammonium chloride, 10 mM potassium bicarbonate and 0.1 mM EDTA) and washed again with 1× PBS. Cells were resuspended in R-10 medium. The differential counting was determined by haematological analyser (XN-1500, Sysmex Europe GmbH). PBMCs from dogs of the field study were frozen in liquid nitrogen until use. PBMCs were cultivated in 24-well plates (2.5 × 10^6^ cells/well) and incubated for 18 h at 37°C in humidified incubator (5% CO_2_) for adherence. Later, cells were washed to obtain (a) only monocytes and RNA was directly extracted or (b) monocytes were treated with 1,25(OH)_2_D at a concentration of 0.1 μM of Lipopolysaccharides from *Escherichia coli* O111:B4 (LPS) (EC N 297-473-0, Sigma-Aldrich) at 0.1 μg/mL as positive control for 24 h. Plates were centrifuged at 400 g for 10 min, washed once with 1x PBS and monocytes were collected with 1 mL TRI Reagent (Ambion) and stored at -80°C until RNA extraction.

### Gene expression analysis of Vitamin D pathway

RNA was extracted from monocytes using the RiboPure RNA Purification Kit (Ambion) following manufacturer’s instructions and measured with a NanoDrop-2000 Spectrophotometer (Isogen Life Science B.V). Retro-transcription was carried out by using High-Capacity cDNA Reverse Transcription kit (Applied Biosystems) following a thermal profile of 25°C for 10 min, 37°C for 120 min, 95°C for 5 min. Levels of *CAMP*, *CBD103*, *CYP24A1*, *CYP27B1*, *NOS2* and *VDR* expression were determined in addition to *RPL18* as a housekeeping gene. To ensure amplification of cDNA sequences derived from retro-transcription of mRNA of interest, primers were designed including exon boundary (Table 1). Amplification of each sample was carried out in triplicate by using SYBR Select Master Mix reagents (Applied Biosystems) with the aid of Applied Biosystems StepOnePlus PCR instrument and StepOnePlus Software v2.3 (Applied Biosystems). The thermal cycling profile was 10 min at 50°C, followed by 40 cycles of 95°C for 10 min, 95°C for 15 s, and 60°C for 1 min. Melting curves assessed the specificity of our amplification products. Cytokine mRNA expression levels were calculated by relative quantification using the 2^−ΔΔCT^ method [41].

**Table 1:** Sequences of primers used for gene expression determinations.

**Gene**		**Primer sequence**	
**CAMP**	F		5’-AGGACACGGGCTACTTTGAC-3’
	R		5’-TTTCGCCAATCTTCTGCCCC-3’
**CBD103**	F		5’-GCCGCTGCTTACTTGTACCT-3’
	R		5’-CCTCATGACCAACAGGCTTC-3’
**CYP24A1**	F		5’-ACTCCTTCGGAAGAATGCGG-3’
	R		5’-CGACCGGGGTTACCATCATC-3’
**CYP27B1**	F		5’-GGCACACCTGACCTACTTCC-3’
	R		5’-AGAGCGTGTTGGATACCGTG-3’
**NOS2**	F		5’-CACAGGATGACCCCAAGTGTC-3’
	R		5’-CAGCTGGCTTGATTGTGGATTC-3’
**VDR**	F		5’-TATCACCAAGGACAACCGCC-3’
	R		5’-CAGGATCATCTCCCGCTTCC -3’
**RPL18**	F		5’-GTCGACATCCGCCACAACAA-3’
	R		5’-AGGTAGAGTTGGTTCGTCTGG-3’

Initials: F and R mean forward primer and reverse primer, respectively.

### Data analysis

In the unadjusted analysis, the comparisons between different groups were performed using unpaired *t*-test and comparisons between same groups but different times using paired *t*-test. For analysis in which distribution does not conform to parametric criterion we used Wilcoxon signed-rank test to compare related samples and Mann-Whitney U test for independent samples. One-way ANOVA was used for multiple comparisons in dose-response experiments. All statistical tests were performed using GraphPad Prism 9.0 software. A *P*-value ≤ 0.05 was considered significant.

## Results

### Vitamin D seasonality in healthy dogs

**Fig 1:**
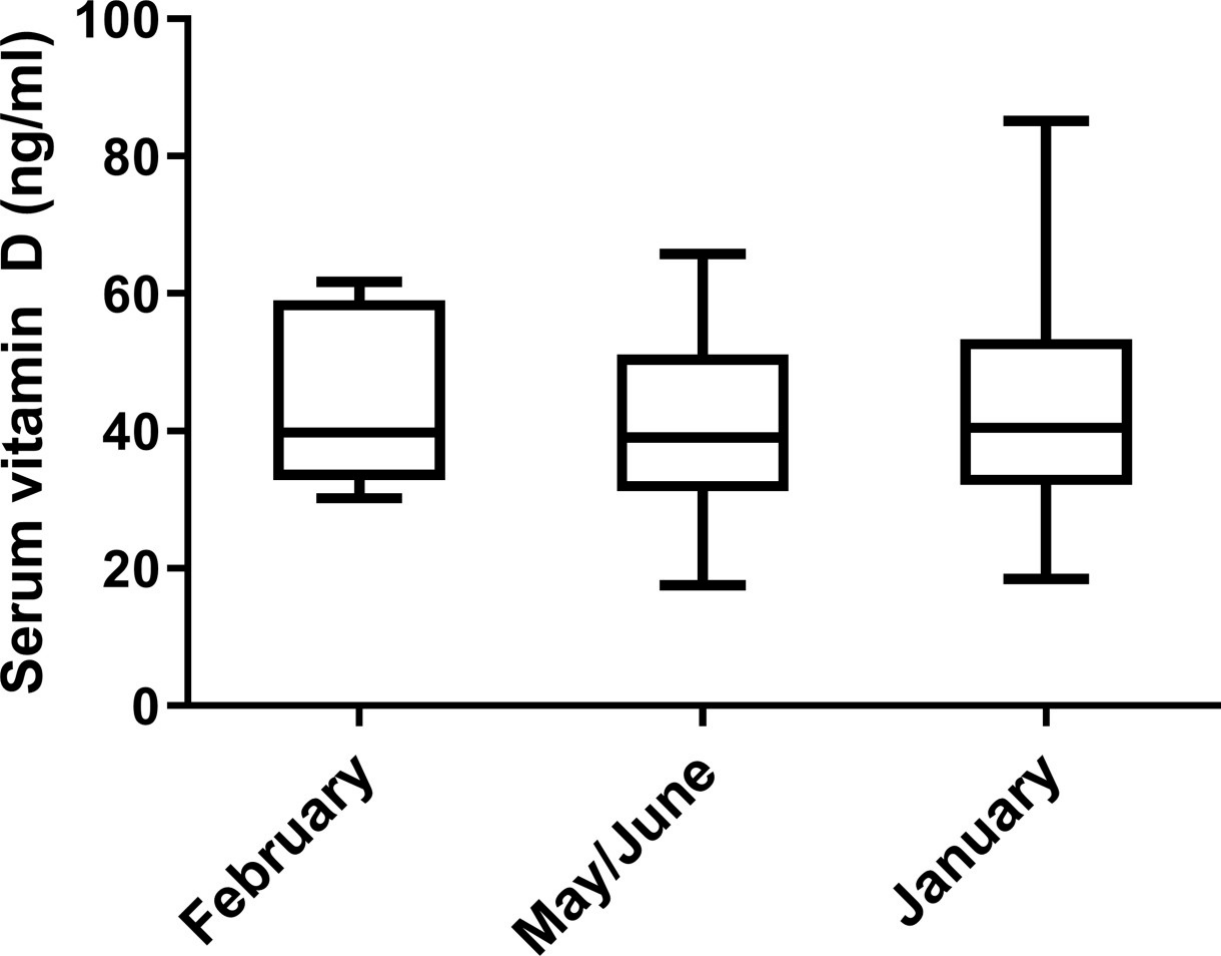
Serum Vitamin D levels of 36 healthy dogs living in Spain at three different time points. Vitamin D status in dogs was assessed according to the serum levels of 25-hydroxyvitamin D estimated with an ELISA test.

The mean 25(OH)D concentration of healthy dogs in the three points evaluated were 44.6 ± 12.5 ng/mL, 41.28 ± 12.4 ng/mL and 43.15 ± 14.8 ng/mL for February, May/June and January, respectively. No statistically significant variation was observed in any of the times studied (Wilcoxon matched-pairs signed rank test) (Fig 1).

### Vitamin D concentration as a risk factor for CanL

We selected 9 *Leishmania*-infected dogs presenting clinicopathological symptoms compatible with this disease (median CPS = 4) and which tested positive for qPCR analysis and/or CTLA-serology at T2 (“Infected” group) and 10 dogs which consistently tested negative in both tests (“Non-Infected” group). The baseline characteristics of both groups at the starting and end points are shown in Table 2.

**Table 2:** Characteristics of the dog population used in the current study at the two points studied.

PARAMETERS	GROUPS OF ANIMALS								Ref. RANGE
	Non-Infected T1		Infected T1		Non-Infected T2		Infected T2		
	Median [IQR]	(+)	Median [IQR]	(+)	Median [IQR]	(+)	Median [IQR]	(+)	
**CPS**	0.0 [0.00–0.00]	0%	0.0 [0.00–0.00]	0%	0.0 [0.00–0.00]	0%	6 [4.00–9.00]	100.0%	≥ 4
**Anti*-Leishmania* Antibodies (EU)**	4.8 [3.55–6.56]	0%	7.8 [7.04–8.41]	0%	5.2 [4.68–5.56]	0%	58.9 [34.91–124.40]	77.8%	≥ 20
**Parasite Load in LN (pp/mL)**	0.0 [0.00–0.00]	0%	0.0 [0.00–0.00]	0%	0.0 [0.00–0.00]	0%	1195.1 [4.5–5862.5]	75.0%	≥ 1

**Fig 2:**
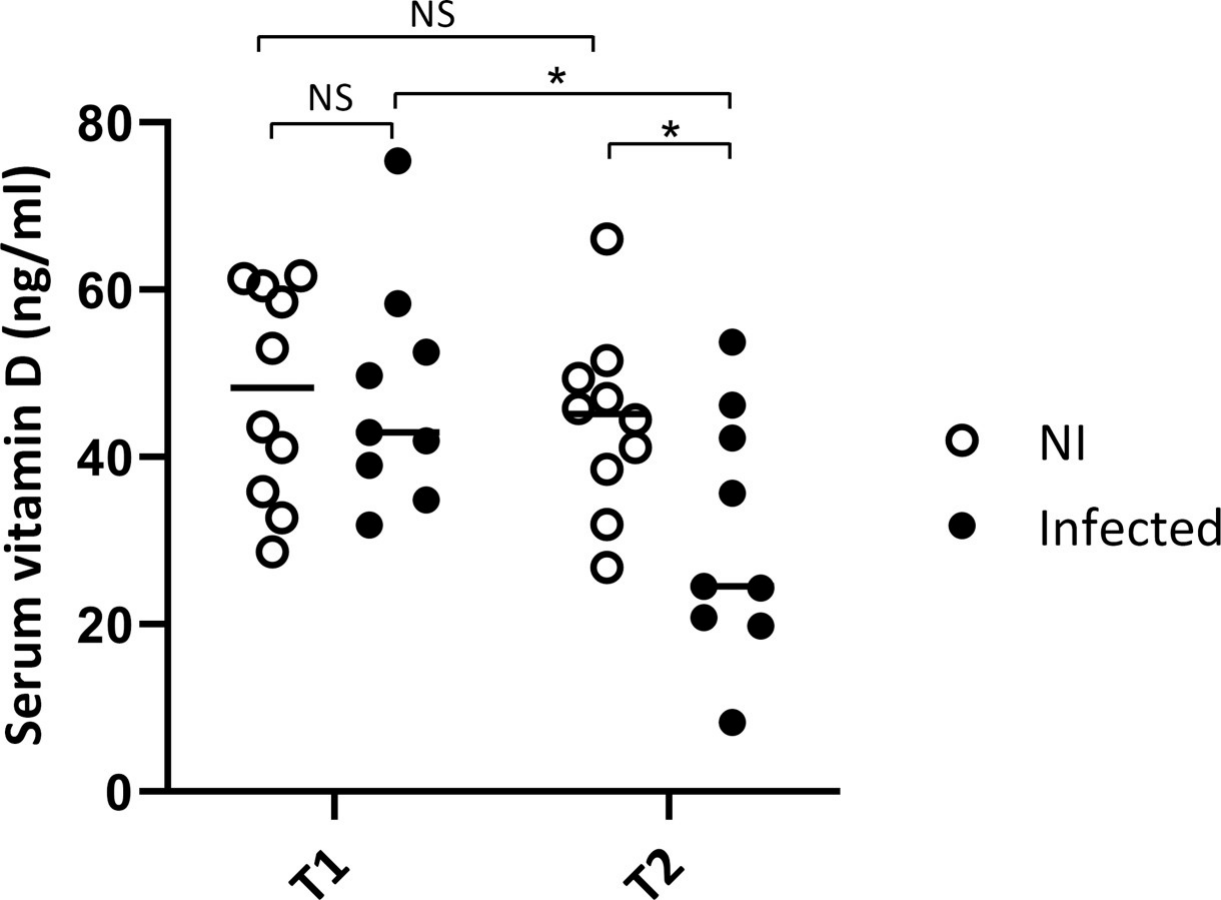
Serum vitamin D concentration in a dog population living in Spain. Vitamin D status in dogs was assessed according to the serum levels of 25-hydroxyvitamin D estimated with an ELISA test. Comparison between Non-Infected dogs (NI) and Infected dogs during the longitudinal study (Infected) at the inclusion point (T1) and at the end of the study (T2) (* *P* < 0.05).

No statistically significant differences in VitD levels were observed between groups at the beginning of study (T1) (unpaired *t*-test; *P* = 0.9619) (Fig 2). Likewise, there were no statistically significant differences between initial and final 25(OH)D levels in the healthy group (paired *t*-test; *P* = 0.1828). Conversely, infected dogs showed statistically significant lower concentration of 25(OH)D in serum at the end of the study (T2) than at the beginning (T1) (paired *t*-test; *P* = 0.0396). At the end of the study, healthy animals showed higher 25(OH)D levels in serum than infected dogs (unpaired *t*-test; *P* = 0.0032). Therefore, sick animals show a greater reduction in VitD (-35.37%) than healthy ones (-7.18%) after a year. The median [interquartile range] levels of 25(OH)D in Non-Infected dogs at the beginning and at the end of the study were 48.3 [37.19–60.00] and 45.2 [39.22–48.80], respectively, and in Infected dogs were 42.9 [39.03–52.57] and 24.5 [20.86–42.27] ng/ml, respectively.

### In vitro effect of vitamin D in *L*. *infantum* parasite killing

**Fig 3:**
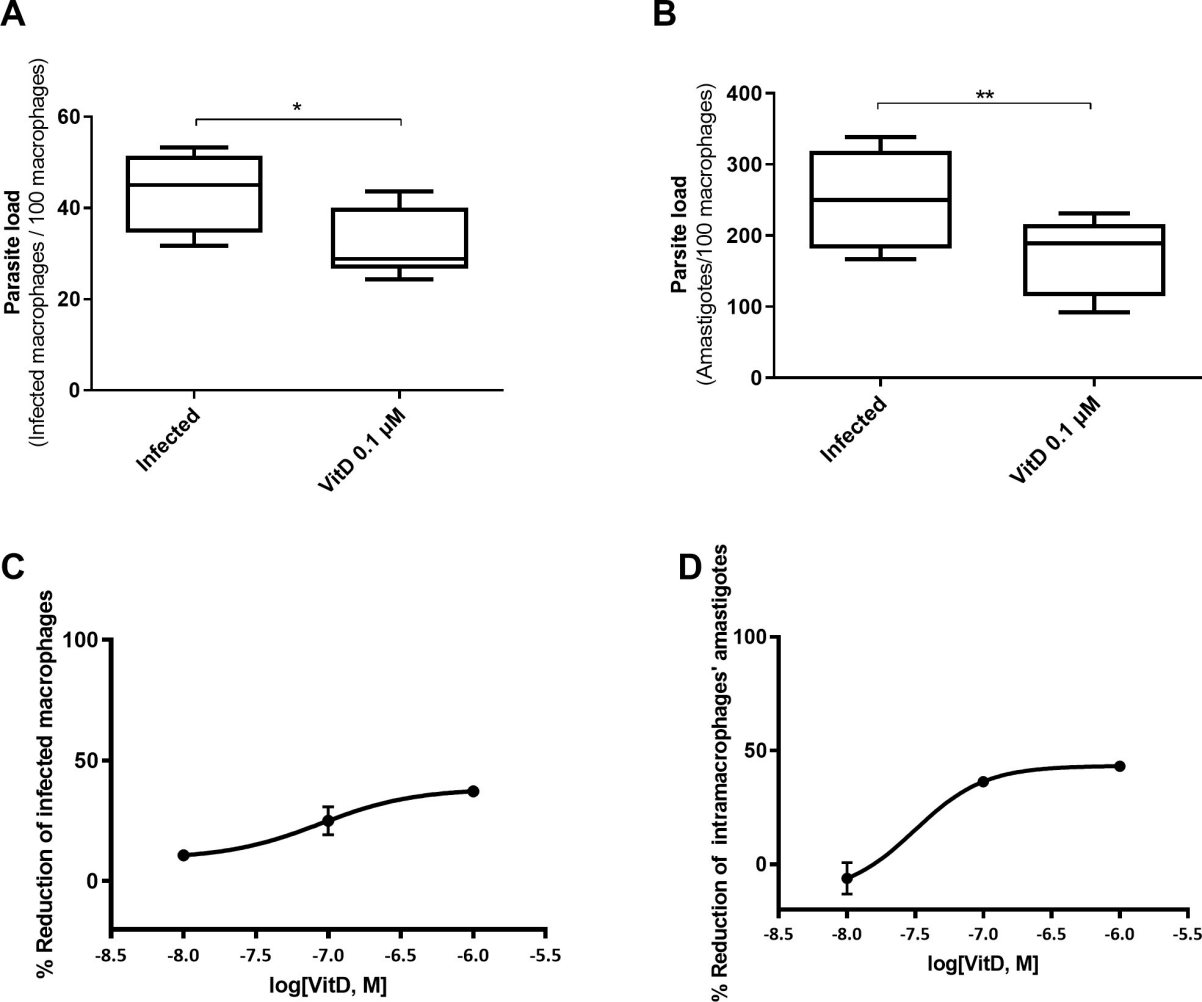
Vitamin D effect on *L*. *infantum* parasite load in macrophages. Number of infected macrophages per 100 macrophages (**A**) and amount of intracellular amastigotes per 100 macrophages (**B**) counted in Giemsa stained-preparations from DH82 macrophages infected with *L*. *infantum* at ratio 5:1 and pre-treated or not (Control) with 1,25(OH)_2_D at 0.1 μM 24 h before infection (* *P* < 0.05, ** *P* < 0.01). Dose response curves showing inhibitory rates of *L*. *infantum*-infected macrophages (**C**) and intracellular amastigote growth (**D**) after 24h of treatment with 1,25(OH)_2_D based on the values for the untreated controls.

In an *in vitro* model using a canine macrophage cell line (DH82) infected by *L*. *infantum* we found that addition of active VitD lead to a significant reduction in parasite load (Fig 3). Pretreatment of canine macrophages cell line with 1,25(OH)_2_D at a dose of 0.1 μM, achieved a reduction of 26.5% in the number of infected macrophages (Wilcoxon matched pairs signed rank test; *P* = 0.0156) and 31.4% in the number of amastigotes per 100 macrophages (Wilcoxon matched pairs signed rank test; *P* = 0.0078) (Fig 3A and 3B). These reductions follow a dose-response effect (ANOVA test; *P* = 0.0285 and *P* = 0.0107, for infected macrophages and number of amastigotes reduction, respectively) (Fig 3C and 3D).

### VitD pathway in primary canine monocytes

**Fig 4:**
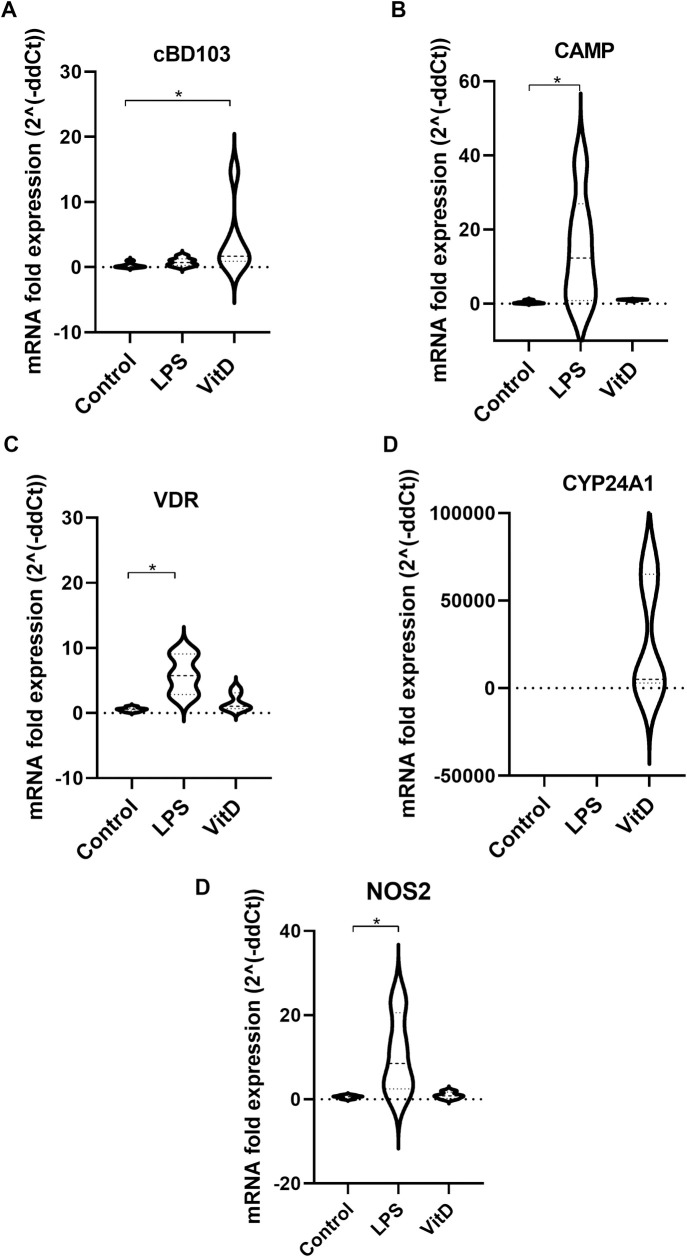
**Fold expression of *CBD103* (A), *CAMP* (B), *VDR* (C), *CYP24A1* (D) and NOS2 (E) genes.** mRNA fold-increase from VitD treated samples was calculated with reference to its negative control (cells of same extraction but untreated). mRNA expression from 0 condition was normalized from a control with a lowest value (* *P* < 0.05).

In a model using monocytes obtained from buffy coat of healthy blood donor dogs from an animal blood bank we found that 24 h treatment with 1,25(OH)_2_D lead to a statistically significant increase of the AMP β-defensin *CBD103* gene compared to the basal expression of untreated monocytes (Wilcoxon signed rank test; *P* = 0.0313). No differences were detected in cathelicidin AMP *CAMP* gene expression (Wilcoxon signed rank test; *P* = 0.0625) neither to the *VDR*, CYP24A1 and *NOS2* gene expression (Wilcoxon signed rank test; *P* = 0.0938 and *P* = 0.0625, respectively) (Fig 4). Addition of LPS at a concentration of 0.1 μg/mL greatly increased *CAMP*, *VDR* and *NOS2* expression (Wilcoxon signed rank test; *P* = 0.0313, both), but not that of *CBD103* and *CYP24A1* (Wilcoxon signed rank test; *P* = 0.0938 and *P* = 0.8125, respectively).

### Gene expression of monocytes from retrospective field longitudinal study

**Fig 5:**
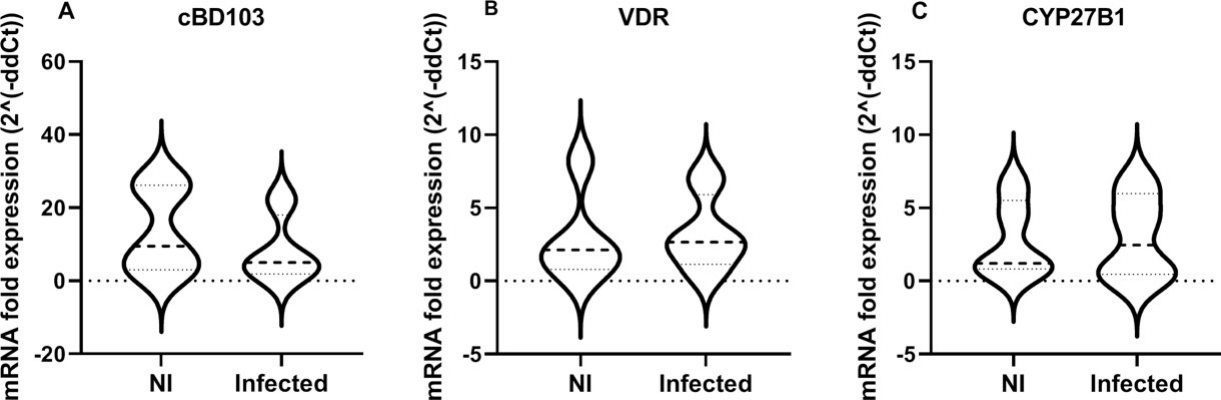
Expression of vitamin D pathway genes. Fold expression of *CBD103* (A) *CYP27B1* (B) and *VDR* (C) genes in Non-Infected (NI) and Infected (Infected) groups at the end of the study (T2).

We have shown that C*BD103* expression increases with the presence of VitD, so that it could play a key role in the antiparasitic activity derived from the action of VitD. For this reason, we analysed mRNA expression of *CAMP*, *CBD103*, *CYP24A1*, *CYP27B1* and *VDR* in monocytes from 9 dogs included in the retrospective longitudinal study from which we were able to collect PBMC samples (4 dogs for Infected group and 5 for the Non-Infected group). We found no statistically significant differences in any of the genes studied. However, results suggest that at the endpoint healthy animals have higher expression of β-defensin *CBD103*, while animals suffering leishmaniasis have higher expression of *CYP27B1*. In the case of *VDR* there are no indications of a differential trend between groups (Fig 5). *CAMP* expression levels were very close to the quantification limit, and reliable results could not be obtained. *CYP24A1* was undetectable.

## Discussion

Our previous study showed that dogs with clinical leishmaniasis presented lower VitD serum concentration than non-infected dogs, and even lower than those with asymptomatic infection [37]. However, it was not possible to prove whether the low VitD levels found in dogs with CanL were the consequence or the cause of this parasitic disease. Although VitD levels have been determined in other canine infectious diseases such as spirocercosis [42], no longitudinal studies have been performed. Based on the literature search, this is the first longitudinal study describing VitD concentration in a canine population living in a highly endemic area of leishmaniasis.

In humans, it has been widely described that VitD status is seasonal due to photochemical activation of VitD in skin by UVB rays [15,43], but it remains unclear if the same is true in dogs because of differences in skin hair. Our results show that VitD concentration in dogs does not follow a seasonal pattern, with similar concentration in winter and spring. These results are consistent with the few previous studies that investigated VitD synthesis in dogs. Whatley and Sher (1961) reported low presence of VitD precursors in the skin of healthy dogs [44] and How et al. (1994) detected a low UV-mediated conversion rate of the precursor 7-dihydrocholesterol to VitD in dogs compared to rats [16]. However, only one longitudinal study has been performed, enrolling huskies from polar latitudes and showing an inverse relationship between UVB radiation and VitD status [45]. This result accounted for the VitD rich diet received by these dogs during the winter. These studies pointed out the importance of VitD supplementation in dogs, but they did not rule out some effect of UVB light on this specie. Our results confirm that VitD status in dogs is not influenced by the number of hours of exposure to sunlight and would not matter the season of the year when assessing VitD levels in clinical practice.

In the present retrospective longitudinal study, we showed that non-infected dogs did not present significant changes in VitD concentration between the beginning and the end of the study one year later, whereas those developing clinical leishmaniasis have a significant VitD decrease at the end of the study (35% reduction). When VitD levels at starting points were compared, no differences between groups were detected. Therefore, VitD concentration could not be used as a prognostic marker of clinical leishmaniasis. However, as VitD concentration decreased with the onset of clinical symptoms, we suggest that VitD concentration could be useful as clinical marker for the evolution of this disease. Low VitD concentration in sick dogs could be a consequence of VitD exhaustion due to the inflammatory process, following a similar pattern as the previously described for vitamin A during chickenpox infection [46]. Decreased VitD with the onset of clinical disease would be consistent with studies that have suggested that low VitD concentration is a marker of ill health [47]. However, we cannot rule out that VitD decrease in *Leishmania*-infected dogs were related to a poor nutritional status, although, only 2 dogs presented a loss of body weight at T2.

One limitation of the present study could be the low number of dogs included in the analysis, but we have to keep in mind that CanL is a disease with a long latent period, and that only 2.5% of dogs is diagnosed for this disease within 12 months in Spain [48]. We are planning future studies including a larger number of animals.

We investigated if VitD plays a role in the control of *Leishmania* load inside macrophages. Our *in vitro* model using a canine macrophage cell line showed that this hormone has a parasite killing activity, since addition of active VitD at 0.1 μM led to a significant reduction in *L*. *infantum* parasite load at 24 h post-infection. This is in line with the inhibitory effect of 1,25(OH)_2_D described for *Toxoplasma gondii* and *Mycobacterium tuberculosis* intracellular growth [18,29] and it suggests that VitD could have a protective *in vivo* effect against *Leishmania*, as it is the case for *Trypanosoma cruzi* infection [49]. The present study and our previous results [37] pointed out that *L*. *infantum* induced VitD deficiency in dogs and at the same time, this deficiency favored parasite dissemination. Other studies investigating the relationship between VitD and response to *Leishmania* infection yielded discrepant results. Ramos-Martinez et al. (2013) reported a significant reduction in the lesion size in *L*. *mexicana*-infected mice treated with 1,25(OH)_2_D [35], while other studies suggest that VitD deficiency increases resistance to *L*. *major* and *L*. *amazonensis* [50–52]. However, these results focused on the cutaneous form and/or in a mouse model, which is predisposed to Th1 immune response. A study with VL patients has shown that people suffering from this disease presented significant lower 1,25(OH)D_3_ serum concentration than healthy people, in agreement with our results [33].

Although the molecules and signals involved in VitD effects against *Leishmania* infection have not yet been investigated, the mechanism of VitD action against tuberculosis infection is well-known. Following TLR-2 activation of human macrophages by *M*. *tuberculosis* antigens, expression of *CYP27B1 and VDR* increases [19]. This ends up in increased expression of AMPs, mainly cathelicidin [12]. AMPs are important innate immunity mediators against microbial pathogens. They act through direct interaction with and disruption of microbial membranes, and indirectly through modulation of host cell migration and activation [53]. There are evidences that mammalian AMP cathelicidin influences control of cutaneous *Leishmania* infection; a *Leishmania* study using a *CAMP* knock-out mouse model showed that the presence of high inflammatory response in infected animals was *CAMP*-dependent [54]. In our retrospective longitudinal study, we did not find statistically significant differences in *CAMP* expression between non-infected and *Leishmania*-infected groups at the end of the study. We also investigated *CAMP* expression in primary canine monocytes as an *ex vivo* model. These experiments also showed no-differences in *CAMP* expression after addition of 1,25(OH)_2_D, even though *CAMP* expression was increase after LPS stimulation. Although in human macrophages 1,25(OH)_2_D increases the expression of *CAMP* directly via VitD response elements in the *CAMP* gene promoter [33,55], the pathway of action of VitD could be different depending on the animal species. In cattle, *CAMP* was not affected by addition of 1,25(OH)_2_D [56]_,_ but it modulates the immune response by increasing NO production in peripheral blood mononuclear cells [57]. On the contrary, addition of 1,25(OH)_2_D did not increase the expression of *CAMP* neither *NOS2* in our *ex vivo* model. For this reason, we investigated other candidates that could explain the parasite killing effect of VitD in *Leishmania*-infected canine macrophages.

The other group of AMPs that can fend off bacterial and viral infections are β-defensins [58,59]. Antimicrobial response of VitD in human macrophages is also mediated by β-defensins through human TLRs [14,60]. In dogs, canine β-defensin 103 (CBD103) has been found in the epidermis of healthy dogs and its expression was altered in atopic animals [61]. In our study, *CBD103* expression was significantly enhanced after 1,25(OH)_2_D addition on primary canine monocytes from blood donors. In addition, we detected that healthy dogs had slightly higher expression of *CBD103* than those suffering from the disease at the end of the study, although this difference was not statistically significant. In agreement with our results, other studies have determined the important role that β-defensins play in host defense against *Leishmania* protozoa [62,63]. In dogs, some SNP’s in *CBD103* gene have been associated with *Leishmania* infection, suggesting that it could be a marker of susceptibility [62]. The expression of β-defensins in *Leishmania*-infected human macrophage cell line THP-1 was induced by the cytokine IL-32γ. The inhibition of IL-32 lead to an increase of *Leishmania* infection index in THP-1 cells whereas its overexpression induced parasite control by AMPs [63]. The detection of IL-32 in canine macrophages would be very useful to determine if β-defensin expression in dogs is also modulated by this cytokine. Functional studies investigating the direct effect of β-defensin on *Leishmania* growth could confirm this molecule as responsible of the observed antiparasitic activity of VitD in canine monocytes.

LPS stimulation did not induce β-defensin expression in canine monocytes. CBD103 has antimicrobial activity against the respiratory pathogen *Bordetella bronchiseptica*, but tracheal epithelial cells stimulated with LPS did not increase β *-*defensins production [64]. Similarly, LPS stimulation was not enough to induce β-defensin expression in cattle monocytes [65]. These authors showed that VitD was the major driver of the β-defensin response of bovine monocytes. These results suggest that VitD pathway in canine macrophages may not be activated via TLR-4, but TLR-2/1 as in humans [66].

After addition of VitD, *VDR* expression remains unchanged in canine macrophages. Treatment of bovine monocytes with the protein translation inhibitor cycloheximide blocked upregulation of β-defensins in response to 1,25(OH)_2_D [65]. This suggests that although β-defensins are targets of 1,25(OH)_2_D in cattle, they are not direct targets of the *VDR*. Nurminen *et al*. (2015) identified multiple transcriptional regulators that are direct targets of VitD in the human THP-1 monocyte cell line. They demonstrated that BCL6 mediated the induction of several of the secondary response genes, and concluded that most of the physiological response of human monocytes to 1,25(OH)_2_D was a secondary response [22]. The same could occur in canine monocytes where we found a significant increase in *CBD103* after adding active VitD but not in *VDR*.

In summary, we have shown that VitD in dogs was not seasonal and was not lower a-priori in dogs that will develop the disease. We have described for the first time the parasite killing activity of VitD addition in *Leishmania*-infected canine monocytes. Our results suggest that this relevant effect could be due to the induction of expression of genes implicated in host defence, such as the AMP β-defensin 103. A future goal derived from this study would be to investigate if VitD or calcitriol supplementation during clinical disease may mitigate the symptoms and progression of this parasitic infection in dogs. Leishmaniases are of great concern because they deeply intertwine pathogenic protozoa, insect vectors, dogs, humans and environment. The "One Health, One World" approach—the interconnection between human medicine, veterinary medicine, environmental science and wildlife conservation—is particularly suited for the control of this kind of diseases. The demonstration that vitamin D can be useful as a clinical marker, and that it decreases parasite load and may have a protective effect in vivo in dogs suggests that it may play a significant role in this holistic approach.

## References

[1] Gállego M. Emerging parasitic zoonoses: Leishmaniosis. OIE Rev Sci Tech. 2004;23(2):661–76. 15702726

[2] Moreno J, Alvar J. Canine leishmaniasis: Epidemiological risk and the experimental model. Trends Parasitol. 2002;18(9):399–405. doi: 10.1016/s1471-4922(02)02347-4 12377257

[3] Badaro R, Jones TC, Carvalho EM, Sampaio D, Reed SG, Barral A, et al. New Perspectives on a Subclinical Form of Visceral Leishmaniasis. J Infect Dis. 1986 Dec 1;154(6):1003–11. doi: 10.1093/infdis/154.6.1003 3782864

[4] Fisa R, Gállego M, Castillejo S, Aisa MJ, Serra T, Riera C, et al. Epidemiology of canine leishmaniosis in Catalonia (Spain): The example of the Priorat focus. Vet Parasitol. 1999 Jun 15;83(2):87–97. doi: 10.1016/s0304-4017(99)00074-6 10392965

[5] Carrillo E, Moreno J. Cytokine profiles in canine visceral leishmaniasis. Vet Immunol Immunopathol. 2009;128(1–3):67–70. doi: 10.1016/j.vetimm.2008.10.310 19054573

[6] Nascimento MSL, Albuquerque TDR, Nascimento AFS, Caldas IS, Do-Valle-Matta MA, Souto JT, et al. Impairment of Interleukin-17A Expression in Canine Visceral Leishmaniosis is Correlated with Reduced Interferon-γ and Inducible Nitric Oxide Synthase Expression. J Comp Pathol. 2015;153(4):197–205. doi: 10.1016/j.jcpa.2015.10.174 26590047

[7] Gonçalves-de-Albuquerque S da C, Pessoa-e-Silva R, Trajano-Silva LAM, de Goes TC, de Morais RCS, Oliveira CN d. C, et al. The equivocal role of Th17 cells and neutrophils on immunopathogenesis of leishmaniasis. Front Immunol. 2017 Oct 30;8:1437. doi: 10.3389/fimmu.2017.01437 29163510 PMC5670345

[8] Bacallar O, Faria D, Nascimento M, Cardoso TM, Gollob KJ, Dutra WO, et al. Interleukin 17 production among patients with American cutaneous leishmaniasis. J Infect Dis. 2009 Jul 1;200(1):75–8. doi: 10.1086/599380 19476435 PMC2732405

[9] Rodríguez-Cortés A, Carrillo E, Martorell S, Todolí F, Ojeda A, Martínez-Flórez A, et al. Compartmentalized Immune Response in Leishmaniasis: Changing Patterns throughout the Disease. Stäger S, editor. PLoS One. 2016 May 12;11(5):1–10. doi: 10.1371/journal.pone.0155224 27171409 PMC4865036

[10] Nylén S, Maurya R, Eidsmo L, Das Manandhar K, Sundar S, Sacks D. Splenic accumulation of IL-10 mRNA in T cells distinct from CD4 +CD25+ (Foxp3) regulatory T cells in human visceral leishmaniasis. J Exp Med. 2007 Apr 16;204(4):805–17. doi: 10.1084/jem.20061141 17389235 PMC2118563

[11] Bhattacharya P, Dey R, Dagur PK, Kruhlak M, Ismail N, Debrabant A, et al. Genetically modified live attenuated Leishmania donovani parasites induce innate immunity through classical activation of macrophages that direct the Th1 response in mice. Infect Immun. 2015;83(10):3800–15. doi: 10.1128/IAI.00184-15 26169275 PMC4567628

[12] Baeke F, Takiishi T, Korf H, Gysemans C, Mathieu C. Vitamin D: Modulator of the immune system. Curr Opin Pharmacol. 2010 Aug;10(4):482–96. doi: 10.1016/j.coph.2010.04.001 20427238

[13] Liu N, Kaplan AT, Low J, Nguyen L, Liu GY, Equils O, et al. Vitamin D Induces Innate Antibacterial Responses in Human Trophoblasts via an Intracrine Pathway1. Biol Reprod. 2009 Mar 1;80(3):398–406. doi: 10.1095/biolreprod.108.073577 19005165 PMC2704027

[14] Wang T-T, Nestel FP, Bourdeau V, Nagai Y, Wang Q, Liao J, et al. Cutting edge: 1,25-dihydroxyvitamin D3 is a direct inducer of antimicrobial peptide gene expression. J Immunol. 2004 Sep 1;173(5):2909–12. doi: 10.4049/jimmunol.173.5.2909 15322146

[15] Jäpelt RB, Jakobsen J. Vitamin D in plants: A review of occurrence, analysis, and biosynthesis. Front Plant Sci. 2013 May 13;4:136. doi: 10.3389/fpls.2013.00136 23717318 PMC3651966

[16] Rabufetti A, Milani GP, Lava SAG, Edefonti V, Bianchetti MG, Stettbacher A, et al. Vitamin D status among male late adolescents living in Southern Switzerland: Role of body composition and lifestyle. Nutrients. 2019 Nov 1;11(11). doi: 10.3390/nu11112727 31717911 PMC6893599

[17] How KL, Hazewinkel HAW, Mol JA. Dietary vitamin D dependence of cat and dog due to inadequate cutaneous synthesis of vitamin D. Gen Comp Endocrinol. 1994 Oct 1;96(1):12–8. doi: 10.1006/gcen.1994.1154 7843559

[18] Hart PH, Gorman S, Finlay-Jones JJ. Modulation of the immune system by UV radiation: More than just the effects of vitamin D? Nat Rev Immunol. 2011 Sep 19;11(9):584–96. doi: 10.1038/nri3045 21852793

[19] Liu PT, Stenger S, Li H, Wenzel L, Tan BH, Wu K, et al. Toll-like receptor triggering of a vitamin D-mediated human antimicrobial response. Science (80-). 2006;311(5768):1770–3. doi: 10.1126/science.1123933 16497887

[20] Jo E-K. Innate immunity to mycobacteria: vitamin D and autophagy. Cell Microbiol. 2010 Aug 1;12(8):1026–35. doi: 10.1111/j.1462-5822.2010.01491.x 20557314

[21] Zughaier SM, Shafer WM, Stephens DS. Antimicrobial peptides and endotoxin inhibit cytokine and nitric oxide release but amplify respiratory burst response in human and murine macrophages. Cell Microbiol. 2005 Sep;7(9):1251–62. doi: 10.1111/j.1462-5822.2005.00549.x 16098213 PMC1388267

[22] Nurminen V, Neme A, Ryynänen J, Heikkinen S, Seuter S, Carlberg C. The transcriptional regulator BCL6 participates in the secondary gene regulatory response to vitamin D. Biochim Biophys Acta—Gene Regul Mech. 2015 Mar 1;1849(3):300–8. doi: 10.1016/j.bbagrm.2014.12.001 25482012

[23] Jeffery LE, Burke F, Mura M, Zheng Y, Qureshi OS, Hewison M, et al. 1,25-Dihydroxyvitamin D 3 and IL-2 Combine to Inhibit T Cell Production of Inflammatory Cytokines and Promote Development of Regulatory T Cells Expressing CTLA-4 and FoxP3. J Immunol. 2009 Nov 1;183(9):5458–67. doi: 10.4049/jimmunol.0803217 19843932 PMC2810518

[24] Tang J, Zhou R, Luger D, Zhu W, Silver PB, Grajewski RS, et al. Calcitriol Suppresses Antiretinal Autoimmunity through Inhibitory Effects on the Th17 Effector Response. J Immunol. 2009 Apr 15;182(8):4624–32. doi: 10.4049/jimmunol.0801543 19342637 PMC2756755

[25] Chen S, Sims GP, Chen XX, Gu YY, Chen S, Lipsky PE. Modulatory Effects of 1,25-Dihydroxyvitamin D 3 on Human B Cell Differentiation. J Immunol. 2007 Aug 1;179(3):1634–47. doi: 10.4049/jimmunol.179.3.1634 17641030

[26] Feldman D, Krishnan A V., Swami S, Giovannucci E, Feldman BJ. The role of vitamin D in reducing cancer risk and progression. Nat Rev Cancer. 2014;14(5):342–57. doi: 10.1038/nrc3691 24705652

[27] Takiishi T, Gysemans C, Bouillon R, Mathieu C. Vitamin D and Diabetes. Endocrinol Metab Clin North Am. 2010 Jun 1;39(2):419–46. doi: 10.1016/j.ecl.2010.02.013 20511061

[28] Ponsonby A-L, Lucas RM, van der Mei IAF. UVR, Vitamin D and Three Autoimmune Diseases—Multiple Sclerosis, Type 1 Diabetes, Rheumatoid Arthritis. Photochem Photobiol. 2005 Nov 1;81(6):1267. doi: 10.1562/2005-02-15-IR-441 15971932

[29] Rajapakse R, Uring-Lambert B, Andarawewa KL, Rajapakse RP, Abou-Bacar A, Marcellin L, et al. 1,25(OH)2D3 inhibits in vitro and in vivo intracellular growth of apicomplexan parasite Toxoplasma gondii. J Steroid Biochem Mol Biol. 2007 Mar;103(3–5):811–4. doi: 10.1016/j.jsbmb.2006.12.058 17270431

[30] Coussens AK, Naude CE, Goliath R, Chaplin G, Wilkinson RJ, Jablonski NG. High-dose vitamin D3 reduces deficiency caused by low UVB exposure and limits HIV-1 replication in urban Southern Africans. Proc Natl Acad Sci U S A. 2015 Jun 30;112(26):8052–7. doi: 10.1073/pnas.1500909112 26080414 PMC4491791

[31] Morris SK, Pell LG, Rahman MZ, Dimitris MC, Mahmud A, Islam MM, et al. Maternal vitamin D supplementation during pregnancy and lactation to prevent acute respiratory infections in infancy in Dhaka, Bangladesh (MDARI trial): Protocol for a prospective cohort study nested within a randomized controlled trial. BMC Pregnancy Childbirth. 2016 Oct 13;16(1). doi: 10.1186/s12884-016-1103-9 27737646 PMC5064894

[32] Cusick SE, Opoka RO, Lund TC, John CC, Polgreen LE. Vitamin D insufficiency is common in Ugandan children and is associated with severe malaria. PLoS One. 2014;9(12):1–8. doi: 10.1371/journal.pone.0113185 25470777 PMC4254466

[33] Das S, Sardar AH, Abhishek K, Kumar A, Rabidas VN, Das P. Cathelicidin augments VDR-dependent anti-leishmanial immune response in Indian Post-Kala-Azar Dermal Leishmaniasis. Int Immunopharmacol. 2017 Sep 1;50:130–8. doi: 10.1016/j.intimp.2017.06.010 28662432

[34] Mukhopadhyay D, Mukherjee S, Roy S, Dalton JE, Kundu S, Sarkar A, et al. M2 Polarization of Monocytes-Macrophages Is a Hallmark of Indian Post Kala-Azar Dermal Leishmaniasis. McMahon-Pratt D, editor. PLoS Negl Trop Dis. 2015 Oct 23;9(10):e0004145. doi: 10.1371/journal.pntd.0004145 26496711 PMC4619837

[35] Ramos-Martínez E, Villaseñor-Cardoso MI, López-Vancell MR, García-Vázquez FJ, Pérez-Torres A, Salaiza-Suazo N, et al. Effect of 1,25(OH)2D3 on BALB/c mice infected with Leishmania mexicana. Exp Parasitol. 2013 Aug;134(4):413–21. doi: 10.1016/j.exppara.2013.05.009 23707346

[36] Bezerra IP da S, Oliveira-Silva G, Braga DSFS, de Mello MF, Pratti JES, Pereira JC, et al. Dietary Vitamin D3 Deficiency Increases Resistance to Leishmania (Leishmania) amazonensis Infection in Mice. Front Cell Infect Microbiol. 2019;9:88. doi: 10.3389/fcimb.2019.00088 31024859 PMC6467002

[37] Rodriguez-Cortes A, Martori C, Martinez-Florez A, Clop A, Amills M, Kubejko J, et al. Canine Leishmaniasis Progression is Associated with Vitamin D Deficiency. Sci Rep. 2017 Dec 13;7(1):1–10. doi: 10.1038/s41598-016-0028-x 28611427 PMC5469782

[38] Rodríguez-Cortés A, Ojeda A, López-Fuertes L, Timón M, Altet L, Solano-Gallego L, et al. A long term experimental study of canine visceral leishmaniasis. Int J Parasitol. 2007;37(6):683–93. doi: 10.1016/j.ijpara.2006.11.007 17239885

[39] Rodríguez A, Solano-Gallego L, Ojeda A, Quintana J, Riera C, Gállego M, et al. Dynamics of Leishmania-specific immunoglobulin isotypes in dogs with clinical leishmaniasis before and after treatment. J Vet Intern Med. 2006 May;20(3):495–8. doi: 10.1892/0891-6640(2006)20[495:doliii]2.0.co;2 16734080

[40] Francino O, Altet L, Sánchez-Robert E, Rodriguez A, Solano-Gallego L, Alberola J, et al. Advantages of real-time PCR assay for diagnosis and monitoring of canine leishmaniosis. Vet Parasitol. 2006 Apr;137(3–4):214–21. doi: 10.1016/j.vetpar.2006.01.011 16473467

[41] Livak KJ, Schmittgen TD. Analysis of Relative Gene Expression Data Using Real-Time Quantitative PCR and the 2−ΔΔCT Method. Methods. 2001 Dec;25(4):402–8. doi: 10.1006/meth.2001.1262 11846609

[42] Rosa CT, Schoeman JP, Berry JL, Mellanby RJ, Dvir E. Hypovitaminosis D in dogs with spirocercosis. J Vet Intern Med. 2013 Sep 1;27(5):1159–64. doi: 10.1111/jvim.12161 23952621

[43] de Oliveira CL, Cureau FV, Cople-Rodrigues C dos S, Giannini DT, Bloch KV, Kuschnir MCC, et al. Prevalence and factors associated with hypovitaminosis D in adolescents from a sunny country: findings from the ERICA survey. J Steroid Biochem Mol Biol. 2020 Jan 1;199:105609. doi: 10.1016/j.jsbmb.2020.105609 32006587

[44] Wheatley VR, Sher DW. Studies of the lipids of dog skin. I. The chemical composition of dog skin lipids. J Invest Dermatol. 1961;36(3):169–70. doi: 10.1038/jid.1961.29 13784747

[45] Griffiths P, Fairney A. Vitamin D metabolism in polar vertebrates. Comp Biochem Physiol—Part B Biochem. 1988;91(3):511–6. doi: 10.1016/0305-0491(88)90014-4 3233926

[46] Campos FA, Flores H, Underwood B A. Effect of an infection on vitamin A status of children as measured by the relative dose response (RDR)13. Am J Clin Nutr. 1987;46:91–4. doi: 10.1093/ajcn/46.1.91 3604975

[47] Autier P, Boniol M, Pizot C, Mullie P. Vitamin D status and ill health: A systematic review. Lancet Diabetes Endocrinol. 2014 Jan;2(1):76–89. doi: 10.1016/S2213-8587(13)70165-7 24622671

[48] Mattin MJ, Solano-Gallego L, Dhollander S, Afonso A, Brodbelt DC. The frequency and distribution of canine leishmaniosis diagnosed by veterinary practitioners in Europe. Vet J. 2014;200(3):410–9. doi: 10.1016/j.tvjl.2014.03.033 24767097

[49] Silva ME, Silva MEC, Silva ME, Nicoli JR, Bambirra EA, Vieira EC. Vitamin D overload and experimental Trypanosoma cruzi infection: Parasitological and histopathological aspects. Comp Biochem Physiol—Part A Physiol. 1993 Jan 1;104(1):175–81. doi: 10.1016/0300-9629(93)90026-z 8094657

[50] Ehrchen J, Helming L, Varga G, Pasche B, Loser K, Gunzer M, et al. Vitamin D receptor signaling contributes to susceptibility to infection with Leishmania major. FASEB J. 2007 Oct 5;21(12):3208–18. doi: 10.1096/fj.06-7261com 17551101

[51] Whitcomb JP, DeAgostino M, Ballentine M, Fu J, Tenniswood M, Welsh J, et al. The Role of Vitamin D and Vitamin D Receptor in Immunity to Leishmania major Infection. J Parasitol Res. 2012;2012:1–10. doi: 10.1155/2012/134645 22007288 PMC3191735

[52] Bezerra JAB, Oliveira IVP de M, Yamakawa AC, Nilsson MG, Tomaz KLR, Oliveira KDS de, et al. Serological and molecular investigation of Leishmania spp. infection in cats from an area endemic for canine and human leishmaniasis in Northeast Brazil. Rev Bras Parasitol Vet. 2019 Nov 4;28(4):790–6. doi: 10.1590/S1984-29612019082 31691733

[53] Lehrer RI, Barton A, Daher KA, Harwig SSL, Ganz T, Selsted ME. Interaction of human defensins with Escherichia coli. Mechanism of bactericidal activity. J Clin Invest. 1989;84(2):553–61. doi: 10.1172/JCI114198 2668334 PMC548915

[54] Kulkarni MM, Barbi J, McMaster WR, Gallo RL, Satoskar AR, McGwire BS. Mammalian antimicrobial peptide influences control of cutaneous Leishmania infection. Cell Microbiol. 2011 Jun;13(6):913–23. doi: 10.1111/j.1462-5822.2011.01589.x 21501359 PMC3121678

[55] Gombart AF, Borregaard N, Koeffler HP. Human cathelicidin antimicrobial peptide (CAMP) gene is a direct target of the vitamin D receptor and is strongly up-regulated in myeloid cells by 1,25-dihydroxyvitamin D3. FASEB J. 2005 Jul 1;19(9):1067–77. doi: 10.1096/fj.04-3284com 15985530

[56] Nelson CD, Reinhardt TA, Thacker TC, Beitz DC, Lippolis JD. Modulation of the bovine innate immune response by production of 1α,25-dihydroxyvitamin D3 in bovine monocytes. J Dairy Sci. 2010 Mar 1;93(3):1041–9. doi: 10.3168/jds.2009-2663 20172224

[57] Waters WR, Miller JM, Palmer M V., Stabel JR, Jones DE, Koistinen KA, et al. Early induction of humoral and cellular immune responses during experimental Mycobacterium avium subsp. paratuberculosis infection of calves. Infect Immun. 2003 Sep 1;71(9):5130–8. doi: 10.1128/IAI.71.9.5130-5138.2003 12933856 PMC187349

[58] Midorikawa K, Ouhara K, Komatsuzawa H, Kawai T, Yamada S, Fujiwara T, et al. Staphylococcus aureus susceptibility to innate antimicrobial peptides, β-defensins and CAP18, expressed by human keratinocytes. Infect Immun. 2003 Jul 1;71(7):3730–9. doi: 10.1128/IAI.71.7.3730-3739.2003 12819054 PMC162002

[59] Kim J, Yang YL, Jang SH, Jang YS. Human β-defensin 2 plays a regulatory role in innate antiviral immunity and is capable of potentiating the induction of antigen-specific immunity. Virol J. 2018 Aug 8;15(1):124. doi: 10.1186/s12985-018-1035-2 30089512 PMC6083524

[60] Liu PT, Schenk M, Walker VP, Dempsey PW, Kanchanapoomi M, Wheelwright M, et al. Convergence of IL-1β and VDR activation pathways in human TLR2/1-induced antimicrobial responses. PLoS One. 2009 Jun 5;4(6):e5810. 10.1371/journal.pone.0005810 PMC2686169 19503839

[61] van Damme CMM, Willemse T, van Dijk A, Haagsman HP, Veldhuizen EJA. Altered cutaneous expression of β-defensins in dogs with atopic dermatitis. Mol Immunol. 2009;46(13):2449–55. doi: 10.1016/j.molimm.2009.05.028 19576634

[62] Da Silva LG, Costa-Júnior CRL, Figueiredo-Júnior CAS, Leal-Balbino TC, Crovella S, Otranto D, et al. Canine β-defensin-1 (CBD1) gene as a possible marker for Leishmania infantum infection in dogs. Parasites and Vectors. 2017;10(1):1–7. doi: 10.1186/s13071-016-1943-1 28427438 PMC5399410

[63] dos Santos JC, Heinhuis B, Gomes RS, Damen MSMA, Real F, Mortara RA, et al. Cytokines and microbicidal molecules regulated by IL-32 in THP-1-derived human macrophages infected with New World Leishmania species. PLoS Negl Trop Dis. 2017 Feb 27;11(2). doi: 10.1371/journal.pntd.0005413 28241012 PMC5344527

[64] Erles K, Brownlie J. Expression of β-defensins in the canine respiratory tract and antimicrobial activity against Bordetella bronchiseptica. Vet Immunol Immunopathol. 2010 May 15;135(1–2):12–9. doi: 10.1016/j.vetimm.2009.10.025 19931188 PMC7112554

[65] Merriman KE, Kweh MF, Powell JL, Lippolis JD, Nelson CD. Multiple β-defensin genes are upregulated by the vitamin D pathway in cattle. J Steroid Biochem Mol Biol. 2015;154:120–9. doi: 10.1016/j.jsbmb.2015.08.002 26255277

[66] Liu PT, Stenger S, Li H, Wenzel L, Tan BH, Krutzik SR, et al. Toll-like receptor triggering of a vitamin D-mediated human antimicrobial response. Science (80-). 2006 Mar 24;311(5768):1770–3. doi: 10.1126/science.1123933 16497887

